# Motility mediates satellite formation in confined biofilms

**DOI:** 10.1038/s41396-023-01494-x

**Published:** 2023-08-17

**Authors:** Mireia Cordero, Namiko Mitarai, Liselotte Jauffred

**Affiliations:** The Niels Bohr Institute, University of Copenhagen, Blegdamsvej 17, DK-2100 Copenhagen O, Denmark

**Keywords:** Microbial ecology, Population dynamics

## Abstract

Bacteria have spectacular survival capabilities and can spread in many, vastly different environments. For instance, when pathogenic bacteria infect a host, they expand by proliferation and squeezing through narrow pores and elastic matrices. However, the exact role of surface structures—important for biofilm formation and motility—and matrix density in colony expansion and morphogenesis is still largely unknown. Using confocal laser-scanning microscopy, we show how *satellite* colonies emerge around *Escherichia coli* colonies embedded in semi-dense hydrogel in controlled in vitro assays. Using knock-out mutants, we tested how extra-cellular structures, (e.g., exo-polysaccharides, flagella, and fimbria) control this morphology. Moreover, we identify the extra-cellular matrix’ density, where this morphology is possible. When paralleled with mathematical modelling, our results suggest that satellite formation allows bacterial communities to spread faster. We anticipate that this strategy is important to speed up expansion in various environments, while retaining the close interactions and protection provided by the community.

## Introduction

While many bacterial species can grow as free-swimming cells in planktonic mode, they will often adhere to a substrate or small enclosure to form dense communities like biofilms [1]. The biofilm community offers protection from local threats. For instance, against the shear flow by attaching to a solid substrate or by protecting the inner cells from the immune system, toxins, or bacteriophages [2, 3]. Also, the local, high cell density helps bacteria share necessary chemicals within the community [4]. The importance of biofilm formation is also reflected in the fact that bacteria have many genes contributing to cell-adhesive structures, such as exo-polysaccharides (EPS), fimbriae, and flagella [5]. These biofilms often grow—as the name indicates—as quasi-two-dimensional (2D) colonies on substrates, but also as three-dimensional (3D) communities in habitats, such as gels, tissues (e.g., the human gut), and soils. Despite this high prevalence, we still lack a full understanding of how dynamic morphologies of 3D biofilms are controlled.

When a bacterial community grows as a dense colony, the surface expands outwards as cells are proliferating, with a doubling time close to what we know from well-mixed liquid cultures. Behind the fast-growing pioneering cells on the front, is a quiescent region, where proliferation is slowed down significantly, due to space constraints [6] and metabolite limitations [7, 8]. Thus, the resulting expansion of the colony surface makes the population grow linearly over time [8, 9], in contrast to the exponential growth in early colonies or liquid cultures. Therefore, the motility of cells is crucial to speed up colony expansion. It is well-established by swimming assays in low density hydrogel (∼0.2% agar), that chemotaxis is enhanced by nutrient shortage [10–12] or attractant gradients [13]. Chemotaxis accelerates expansion dynamics of populations by allowing access to more nutrient [14]. In liquid media, the chemotaxis of the model bacterium *Escherichia coli* is driven by swimming, specifically bundling and propelling of flagella, which gives rise to a run-and-tumble motion. In visco-elastic media, bacterial migration is restricted more and more as elasticity is raised and pore size diminished [15], and the chemotaxis has been reported to be reversely proportional to the elasticity of the medium [12]. Therefore, above a certain threshold of medium density, colony expansion is solely growth driven. Furthermore, in a dense hydrogel (*>*0.5% agarose), the stress at the interface (between colony and media) gives rise to internal ordering of cells [16]. But what happens in the intermediate regions, where the hydrogel is semi-dense? Is it possible that cells can both retain colony characteristics but still speed up the expansion to enhance colonization?

Here we use mono-clonal *E. coli* colonies embedded in an agarose matrix [2, 17] as simple models of 3D colony formation. We use a combination of experiments and mathematical modelling to show how 3D colonies can join growth and flagella-based motility to colonize their local environment. We investigate how the spreading depends on the expression of extra-cellular structures and hydrogel concentration. We demonstrate that indeed 3D colony expansion—driven by the combination of growth and motility—gives rise to satellite colony formation and acceleration of the population expansion to be super-linear over time.

## Results

### Satellite colonies form around 3D colonies

To mimic 3D biofilm evolvement in natural settings, *E. coli* MG1566 wild-type (wt) single cells were embedded in low concentration (≈10 cells/ml) in a semi-dense agarose (0.3%) matrix and incubated for 15 h at 37°C in a minimal defined medium composed of M63 supplemented with 20 μm/ml glucose (M63 + glu). This protocol (Materials and Methods) resulted in confined, mono-clonal quasi-spherical colonies on the order of a few hundred μm in diameter. We also verified that the colony expansion after 15 h has slowed down from exponential to become linear over time (Supplementary Fig. S1). In parallel, we performed a similar experiment using a rich Luria-Bertani (LB) medium and a shorter incubation time of 13 h to account for differences in doubling time in the two media: 27.8 min (LB) vs. 68.4 min (M63 + glu) (Supplementary Fig. S2). In similar studies, these has been referred to as 3D biofilms [16, 18, 19], but here we use the term colony.

**Fig. 1:**
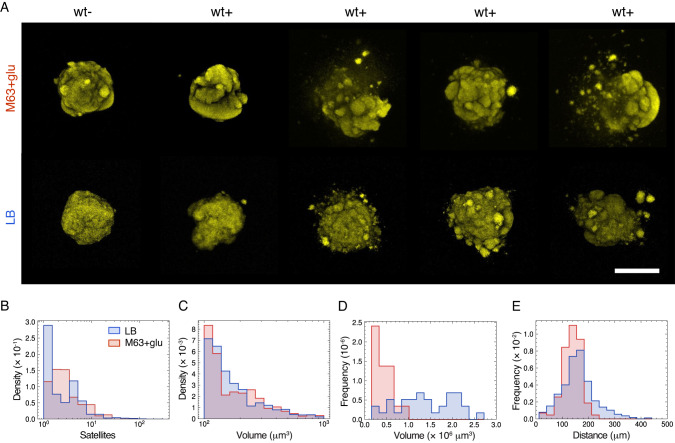
Satellite morphology in mature 3D colonies. **A** Examples of pseudo-colored 3D colonies (maximum intensity projections) in 0.3% agarose and minimal (M63+glu) or rich medium (LB). These examples show inhomogeneity of morphologies without satellites (wt−) or with satellites (wt+). The scale bar corresponds to 200 μm. **B** Distributions of the number of satellites pr. colony (log-scale) in either LB (*N* = 17) and M63 + glu (*N* = 34). **C** Distributions of satellite volumes (log-scale) in either LB (*N* = 149) or in M63 + glu (*N* = 574). **D** Distribution of volumes of the main colonies in either LB (*N* = 17) and M63+glu (*N* = 34). **E** Distributions of distances from the center-of-mass of satellites to the center-of-mass of the main colony in either LB (*N* = 17) and M63 + glu (*N* = 34).

The resulting colonies grew as compact quasi-spherical colonies with a mountainous surface of multiple protrusions, denoted as wt− (Fig. 1A, first column). However, for the majority of 3D colonies, 34/40 (M63 + glu) and 17/18 (LB), these protrusions seem to have escaped the colony as small stationary satellite colonies, denoted as wt+ (Fig. 1A). Both morphology types occurred for colonies grown in both the minimal (M63+glu) and the rich media (LB). It is worth noticing that these two morphologies were not determined by any obvious variations between experiments, as we found wt− and wt+ side by side in the same culture wells. To evaluate whether this behavior was dependent on the specific hydrogel, we did a similar assay interchanging agarose with agar (0.5%) and found the majority of 3D colonies, 21/39 (M63 + glu) to be wt+ (examples in Supplementary Fig. S3).

From the subset of 3D colonies with satellite morphology (wt+), we measured the distributions of number of satellites, their volumes, distances to the main colony, as well as the main colony volumes, for both minimal (blue) and rich (red) medium (Fig. 1B–E). The distribution of the number of satellites per 3D colony (Fig. 1B) shows that the majority had less than 10 satellites. Furthermore, from the distribution of satellite volumes, we found that satellites with smaller volumes had higher prevalence (Fig. 1C). The typical satellite size (*<*10^3^ μm^3^) was about three orders of magnitude smaller than the average volumes of the main colonies, which were (1.3 ± 0.6)·10^6^ μm^3^ (M63 + glu) and (4 ± 1)·10^5^ μm^3^ (LB) (Fig. 1D). Finally, the distributions of distances between center-of-masses of the satellites and their respective main colony had average distances of 161 ± 64 μm and 137 ± 35 μm for minimal (M63 + glu) and rich (LB) medium, respectively (Fig. 1E). Our definition of a satellite is that it is completely detached from the main colony. It is, therefore, possible that we wrongly exclude some of the smaller (<100 μm) distances between the center-of-masses (Fig. 1E), as the satellites have partly merged with the main colonies. From time-lapse imaging we found incidences of satellites forming and subsequently merging with the main colony (Supplementary movies 1–5).

A possible mechanism behind satellite formation is the combination of bacterial cell-cell interaction, motility, and the growth of the cells: A few cells can move away from the surface of the main colony escaping from their attachment to migrate to a different position in the gel, and each of them starts to grow into a new satellite colony. Therefore, in the following, we investigate how bacterial surface structures, as well as the density of the substrate, affect the emergence of satellite colonies. The first is achieved by studying mutants with deletions of structures important for adhesion and motility, and the latter by modifying agarose concentration in the extracellular matrix.

### Deletion of extracellular structures causes loss of satellite morphology

**Fig. 2:**
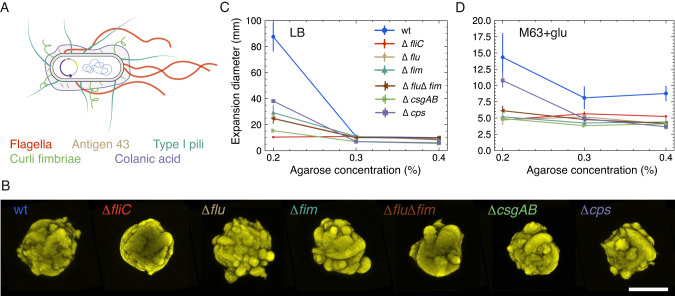
3D morphology and motility of knock out mutants. **A** Sketch of parental (wt) *E.coli* strain (not to scale). The sketched elements include the genome, the fluorescence-carrying plasmid and the extracellular structures: flagella (red), antigen 43 (beige), type I pili (dark green), curli fimbriae (light green), and colanic acid (purple). **B** Morphology of mutant 3D colonies. Examples of pseudo-colored fluorescent colonies (maximum intensity projections) grown in M63 + glu with 0.3% agarose. Scale bar corresponds to 200 μm and color coding is the same as in (**A**). Swimming assay in low density agarose. Diameters of expansion zones after 24 h of incubation vs. agarose concentrations in LB (**C**) and M63 + glu (**D**).

One of the factors governing the morphology of 3D colonies and the appearance of satellites must be the complex interplay between cell envelope components and the extracellular matrix. So, bacterial cell-adhesive structures, such as exo-polysaccharides (EPS), fimbriae, and flagella are obvious components that can modulate the colony morphology. Therefore, we investigated a collection of mutants lacking diverse bacterial surface structures (Table 1). Figure 2A sketches the parental strain (wt) and the suppressed structures: flagella, type I pili, colanic acid, curli fimbriae and antigen 43.

**Table 1:** *Escherichia coli* strains used in the study.

Name	Strain	Relevant characteristics	Reference
wt	MS613	MG1655 K-12 reference (F-lambda- ilvG- rfb-50 rph-1) strain	[62]
∆flu	MS427	MG1655 ∆flu	[68]
∆fim	MS428	MG1655 ∆fim	[24]
∆flu∆fim	MS528	MG1655 ∆flu,∆fim	[24]
∆cps	RMV340	MG1655cps::tet	[69]
∆csgAB	RMV612	MG1655csgAB::kan	[63]
∆fliC	NM109	MG1655fliC::kan	This study

To examine the influence of these surface components, we examined the 3D colony morphology after knock-out deletion and compared it with the wt colony morphology (Fig. 2B).

Flagella are helical filaments responsible for some of the locomotion mechanisms adopted by bacteria. By expressing flagella, cells can self-propel in liquids (swimming) and on surfaces (swarming) [20]. Furthermore, flagella have been shown to play a role in cell–substratum (e.g. initial colony formation [21]) and cell-cell interactions [5]. However, the role of these structures in bacterial motility in confined environments remains largely unknown [22]. We tested a Δ*fliC* mutant and did not find any satellite formation (*N* = 21). We also assessed a Δ*fim* mutant without type I pili, which is a hair-like structures crucial for cell-cell adhesion [21]. The morphology of these mutants was also indistinguishable from that of wt− (*N* = 21).

The Δ*flu* mutant has a deletion in the gene encoding the auto-transporter protein antigen 43, which comes in high copy numbers (up to 50,000 pr. cell [23]). Antigen 43 favors cell-cell interactions through chain-formation and is referred to as a *handshake* protein [24–26]. Given the inverse regulation of pili and antigen 43 [27], we tested both Δ*flu* (*N* = 21) and the double mutant Δ*flu*Δ*fim* (*N* = 22) and found that both display a loss of satellite morphology; even when agarose was interchanged with 0.5% agar (Supplementary Fig. S4).

We also tested the EPS colonic acid mutant, Δ*cps* (*N* = 17), as well as the Δ*csgAB* (*N* = 18) with a deletion in the gene encoding a fibrous surface protein, curli fimbriae, important for both cell-cell and cell-substrate adhesion [28–32]. Even though *cps* and *csgAB* predominantly are expressed at ambient temperature [30, 33], the wt has in a prior been found to have capsules in contrast to the EPS colonic acid mutant (Δ*cps*), which had none [34]. In accordance with that we found wt+ morphology to be lost in both cases. In summary, we found that the satellite morphology (wt+) is lost in the confined colonies of all tested mutants.

### Deletion of extracellular structures reduces motility

Except for the flagella deletion that disables the most common mechanisms of bacterial motility (swimming/swarming), the effect of the other tested surface structures on bacterial confined motility is less apparent. To investigate this further, we performed classical motility assays in low density agarose for all strains used in this study. The assay was done using both rich (LB) and minimal media (M63 + glu) with varying agarose concentrations (0.2–0.4%).

The parental strain (wt) was the most motile in the low density (0.2% agarose) rich medium (LB), with an average distance reached of about three times the one of the second fastest, Δ*cps* (Fig. 2C). All strains were found to be motile except the non-flagellated mutant, Δ*fliC*, but with large variations in speed. This is in accordance with the fact that many of the bacterial surface structures display interdependent regulation [35]. At higher substrate stiffness (0.3–0.4% agarose), where swimming is no longer the main driver of motility, the average moving distances were considerably reduced for all strains.

We also tested all strains’ behavior in a minimal medium (M63 + glu) (Fig. 2D). In the low density minimal agarose (0.2%) the motility of all strains was reduced, as can be seen by comparing the maximum distance reached by wt: (14.3 ± 3.7) cm vs. (87.5 ± 11.4) cm in rich medium (LB). The reduced motility in minimal media is consistent with previous studies: it has been attributed to the considerable metabolic cost of producing flagella [36, 37], while a recent study [13] has shown that the chemotaxis is strongly reduced when there is no supplement attractant, even if the primary carbon source is also an attractant. Again, at greater agarose concentrations (0.3–0.4% agarose) motility was reduced to a non-detectable level for all other than the wt.

Overall, the low density agarose assay suggested that all the mutant strains tested here have reduced motility compared to the wt strain. This is in line with the view that the satellite colonies form as a compromise between their inherent motility and the mechanical constraints imposed by the environment.

### Density of extra-cellular matrix controls satellite emergence

Another factor that affects both bacterial motility and colony morphology is the density of the enclosing matrix. To explore the colony morphology dependence on this environmental factor, we limited our analysis to a comparison between the highly motile wt and the non-flagellated mutant (Δ*fliC*) that was non-motile in all tested experimental settings. We investigated mono-clonal colonies of both strains in varying agarose concentration (0.25–0.35%) in rich (LB) and minimal (M63 + glu) medium.

**Fig. 3:**
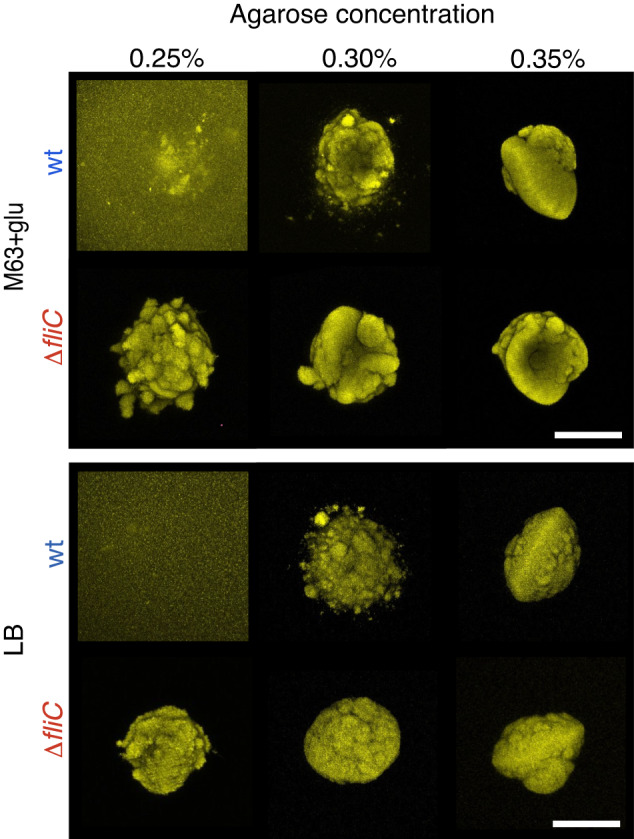
Effect on matrix density on 3D colony morphology. Examples of pseudo-colored fluorescent parental (wt) or flagella mutant (Δ*fliC*) colonies (maximum intensity projections) grown in either minimal (M63+glu) or rich (LB) medium at various agarose concentrations. The scale bar corresponds to 200 μm.

As already mentioned, we found satellite morphology (wt+) among the 3D colonies in 0.3% agarose with frequencies of 34/40 and 17/18 for the wt strain in minimal and rich medium, respectively (Fig. 3). Namely, the satellite morphology was conserved despite changes in nutrient composition and possible slight changes in elasticity between the experimental samples [38].

When reducing the agarose concentration (0.25%) the distinct satellite morphology was lost for wt, and general spreading of the cells inside the gel was observed (Fig. 3, 0.25%). On the other hand, when stiffness was increased (0.35%), 3D colonies grew under greater confinement, resulting in the loss of wt+ morphology. In contrast, the non-motile strain (Δ*fliC*) always grew as single compact colonies at all tested agarose concentrations. We still observed the agarose concentration dependence on the morphology, as at 0.25% agarose concentration the surface of Δ*fliC* colonies appeared more loosely connected than at higher agarose concentrations.

Colony surfaces became smoother as matrix stiffness increased for both strains (Fig. 3, 0.35%). In some cases

(7/18 in M63-glu and 6/9 in LB), we even found slightly oblate colony shapes. This shape change depends on the density of the environment, such that for higher stiffness (*>*0.4% agarose) all tested 3D colonies grew as single oblate colonies (Supplementary fig. S5), in agreement with earlier reports [39]. Recently, this shape morphology has been suggested to originate from the stiffness contrast between the colonies and the environment [16]. These findings all together suggest that the emergence of satellites is a transitional state between the morphologies: i) where cells can swim—more or less freely—through the extra-cellular matrix, and ii) where cells are strictly confined.

### Satellite formation possibly speeds up colonization

The overall experimental result suggests that the observed satellite morphology is due to a combination of local growth of confined colonies and rare excursions of motile cells. Therefore, we propose a *simple* mathematical model of colony growth that has these two features and show that such a model indeed reproduces the 3D morphology of the confined bacterial colonies. We then used this model to further study how this mode of expansion can contribute to the overall colonization.

For this purpose, we modified the well-known Eden growth model, which is a lattice model, where the surface cells can divide to occupy empty nearest neighbor sites [40, 41]. Our modification allows for occasional escapes of single cells from the main colony. In other words, surface cells do not only grow but can also migrate to another empty site in the lattice. We assumed that the cells swim from site to site on timescales much faster than the time between two cell divisions (25–75 min, Supplementary Fig. S2). This assumption is based on the fact that cell divisions will decelerate swimming and experimental findings for *E. coli* in porous media [15]. So instead of simulating the cells’ actual trajectory, we implemented the dispersal as a jump to a final position; chosen randomly by a radial Gaussian probability distribution centered around the starting site. Therefore, the model is essentially controlled by two parameters. The first is the rate of jumps, *k*_*s*_, when the cell doubling rate, *k*, is set to unity. The second is the standard deviation, σ, of the Gaussian distribution of possible new sites to jump to. We ran the simulation until a million jump/division events. Thus, the resulting in silico colonies had widths of about 100 μm assuming i) colonies are compact and ii) one occupied lattice site corresponds to one cell volume (∼1 μm^3^).

3D in silico colonies were obtained with *σ* ∈ {2,5,7,10} and *k*_*s*_ ∈ {0.001,0.05,0.1,0.2}. At low σ and *k*_*s*_, the colonies were compact with smooth surfaces, as the distances (σ) rise, small protrusions appear on the surface followed by satellite breakouts (Supplementary Fig. S6). As anticipated, the number of satellites also increased as the jump frequency (*k*_*s*_) increased and ultimately (*σ* = 10 and *k*_*s*_ = 0.2) the simulated system was no longer confined. Instead, the cells spread mimicking the experimental results of wt in the low density agarose assay (Fig. 3, 0.25%).

**Fig. 4:**
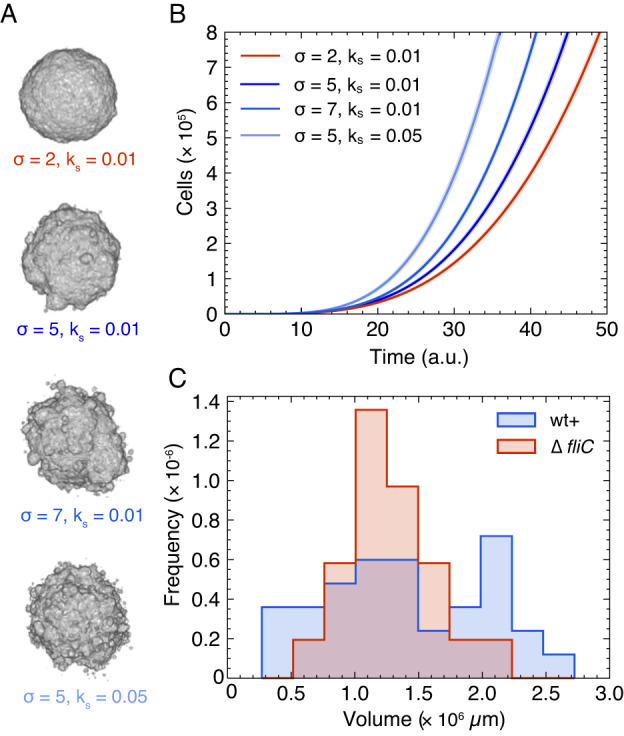
Population size and time evolvement. **A** Effect of frequency, *k*_*s*_, and distance, *σ*, of jumps in the simulations of 3D colonies after 10^6^ division/jump events; corresponding to motile (blue) and non-motile (red) colonies. **B** Time-evolution of each of the four examples (*N* = 25) given in (**A**), where time is defined as detailed in Materials and methods. The shaded region corresponds to ±SD. **C** Distribution of total colony volumes in minimal (M63+glu) medium of wt+ (*N* = 34) and the flagella mutant Δ*fliC* (*N* = 21). The wt+ corresponds to the sum of volumes in Fig. 1C+D (M63 + glu).

In this system, the smooth and compact colonies (Fig. 4A, red) mimic the non-motile Δ*fliC* colony (Fig. 2B) and the others (Fig. 4A, blue) different wt colonies (Fig. 1A). We analysed our in silico 3D colonies (*N* = 30 of each) in the exact same way as in the experimental data (Fig. 1B–E) and found similar trends (Supplementary Fig. S7). With this we explored time evolution (Fig. 4B) and found that the colonies with wt+ morphology (*σ* = 5 and *k*_*s*_ = 0.01, *σ* = 7 and *k*_*s*_ = 0.01, and *σ* = 7 and *k*_*s*_ = 0.01) clearly grew faster than the compact colonies (*σ* = 2 and *k*_*s*_ = 0.01). Hence, these results predict a view, where satellite outbreaks speed up the colonization of the extra-cellular matrix.

To test this prediction, we compared the experimental distributions of final total volumes of wt+ (blue) and Δ*fliC* (red) (Fig. 4C). We found that the occurrence of satellites results in a more widespread distribution of volumes compared to the non-motile strain (Δ*fliC*). Surprisingly, there were also wt+ colonies with smaller volumes even though their doubling time may be slightly faster than that of Δ*fliC*: 68.4 min vs. 72.9 min (Supplementary Fig. S2). While the wide distribution makes it hard to compare the average values in the current sample size, the wider distribution with more satellite colonies is in qualitative accordance with the distributions of the simulated 3D colonies (Supplementary Fig. S8).

## Discussion

Even though the flagella-mediated motility of *E. coli* is well-described by the run-and-tumble model [42], we still lack the full understanding of how bacteria migrate through porous or visco-elastic media. Several theoretical works concerning flagella-based motility describe how the migration can be slowed down when colliding with structures of various sizes [43–45]. Moreover, in a recent study, the individual trajectories of bacteria in a porous medium were imaged and the velocity was found to be highly dependent on pore size [15]. Furthermore, agarose gels have “spongy” microstructures with a broad distribution of pore sizes. Particularly, for the semi-dense agarose (0.3%) used in this study, the pores range from a few hundred nanometers to a few micrometers (see ref. [46] Fig. 16 for a metastudy). Relating this to our results, the porous hydrogel will barely allow the bacteria to squeeze through. Altogether, these reports indicate that *E. coli* moves through a porous medium constantly alternating between stalling (being trapped), re-orientation, and swimming. In accordance with this, we find that flagella are necessary for migration through the various semi-dense media used in this study (Fig. 3). However, the behavior we observed is still very different from swimming in agarose (Fig. 2C+D) as the main colonies (as well as the satellites) in 0.3% agarose had sharply defined surfaces, indicating that cell outbreak is a rare event. Also, we did not see any signs of the collective colony detachments, recently reported in mature colonies [19, 47, 48].

Given these observations, we propose that the satellite colonies are formed by the rare—but not too rare—detachment and migration of a single surface cell, followed by stalling, and subsequent growth. So, the volumes of satellites indicate when, in the colony’s history, the excursion occurred. Furthermore, the size also tells us that the dispersal of the founder cell was long enough to allow the sub-cluster to form before it is annexed to the main colony. We observed that larger satellites (*>*500 μm^3^) were much less common (Fig. 3 wt LB 0.3% for an example) than smaller ones (Fig. 1B), which is consistent with the proposed mechanism.

Our lattice model, which is based on an Eden growth model with rare jumping events added, successfully produced 3D colonies with satellite morphologies in a specific parameter range. The model predicted that the distribution of total colony volumes would be more widespread when the satellite formation is more frequent (e.g. compare *σ* = 5 and *k*_*s*_ = 0.05 vs. *σ* = 2 and *k*_*s*_ = 0.01 in Supplementary Fig. S8). This is consistent with our experimental findings, where we find the distribution of total volumes of the flagella mutant (Δ*fliC*) to be much narrower than for the parental (wt) strain (Fig. 4C).

Hallatscheck and co-workers [49, 50] have theoretically demonstrated the impact of long-range dispersal in population expansion, especially when the dispersal distance distribution has a somewhat fat tail. In our model, the distribution of dispersion distances is Gaussian, but still the expansion dynamics of satellite-forming colonies is indeed faster than linear (Fig. 4B). On the other hand, it is also significantly slower than exponential growth (Supplementary Fig. S2). So, satellites form and then merge with the main colony and, thereby, limit the overall growth of the population. Nevertheless, our model suggests that in long term, the system will keep growing super-linearly, as the number of jumps (which is equivalent to the initialization of satellites) scales with the colony surface. In other words, as the growth of the main colony is linear (Supplementary Fig. S1) and the distribution of migration distances is unchanged over time, new satellites will keep forming and the main colony will never catch up with all the satellites. However, our model does not encompass spreading strategies on very long timescales, as the model ignores the effects of nutrient depletion and time-varying jumping distributions.

In our experiment, some of the main colonies of mutants produced very rough surfaces. Whilst the flagella knockout mutant’s surface (Δ*fliC*) was overall smooth, the other mutants had several small protrusions of their surfaces (Fig. 2B). There are a few factors that can possibly contribute to the roughness of the surfaces. The first is that even for the mutants, which are less motile than wt (Fig. 1C+D), individual surface cells escape occasionally. However, they travel significantly shorter distances than wt cells do. If this is the case, the satellites will quickly be re-absorbed into the main colony (Supplementary videos 1–5). In other words, the mountainous surface could be explained by motility with short dispersals (small σ). This is also in accordance with the fact that higher agarose concentrations (*>*0.3%) resulted in smoother surfaces (Fig. 3). Another possibility is the nutrient driven instability of the surface growth. Several studies have shown how surface roughening instabilities are reinforced by the resulting local nutrient depletion [51]. The nutrient-driven instability also explains the (pseudo-fractal) broccoli-like morphology recently reported for 3D *E. coli* colonies [52]. It is possible that such instabilities are relevant, especially in the later stage of colony development. However, this effect is not included in our model since it does not consider space limitations and nutrient depletion separately.

Whilst the model compares well with our experimental results with respect to the distributions of satellites, main colony volumes, and distances to satellites (Fig. 1C–E vs. Supplementary Fig. S7B–D), it fails to reproduce the distributions of the number of satellites (Fig. 1B vs. Supplementary Fig. S7A). We conclude that with only two parameters (*σ* and *k*_*s*_), we cannot cover the varieties of the natural system or the rare successful escapes. We speculate that this could be related to the variance in flagella abundance, even among cells with the same genotype. It is well-known that the expression of flagella is highly heterogeneous, particularly that there are large stochastic variations in number of flagella. This has been suggested to be an evolutionary favorable strategy, as the expression of flagella is very energy consuming [53], such that motility is retained on colony level even when motility is limited on a cellular level.

Except for the mutant that did not express flagella, the studied strains lacked structures related to adhesion and aggregation processes rather than to motility. Still, we do not fully understand why the antigen 43 mutant (Δ*flu*), is less motile than the wt and—maybe as a result—hindered in satellite formation (Fig. 2). Biofilm formation has been shown to be associated with the expression of antigen 43 [24] and type I pili [21]. So one might assume that antigen 43 repression would up-regulate motility. However, the picture is more complex, as both EPS colonic acid [34] and type I pili [27] block antigen 43 function. Moreover, both antigen 43 and type I pili expressions are phase variable [54] and they are inversely regulated [55]. Therefore, more studies must be done to unravel the role of the surface structures per se, without the inverse regulations, phase variability, and physical shielding.

In general, dominant aggregation phenotypes are expected to hinder motility. Hence, it is reasonable to expect that when these processes are suppressed, the motility will increase. However, in our experiments, all the mutants presented reduced motility compared to their parental strain. This demonstrates the complexity of the bacterial motility phenotype. Indeed, vast amount of literature indicate the complex interdependence of motility and surface structure genes. Over-expression of the *handshake* protein antigen 43 (*flu*) has been reported to impair motility, not because of increased aggregation but due to interfering with the expression of flagella [56]. Similarly, the constitutive expression of type I fimbriae (*fim*) compromises the motility of bacteria by reducing the expression of flagellin [57]. The expression of curli (*csgAB*) and colanic acid (*cps*) are promoted by regulatory networks that down-regulate motility [58–60], therefore they are normally expressed in a complementary manner. Similar motility has been reported between wt and Δ*flu* [56], and between strains with and without type I fimbrial expression [61], while this was not the case in our system (Fig. 2).

Taking our results together, we suggest that satellite-formation allows bacteria to colonize unknown horizons faster (super-linear over time), whilst still retaining the tight bindings of the biofilm. Such spreading behavior can be advantageous, when invading complex environments, such as competing microbial communities, soils, or mammalian tissues. While we observed the phenomenon in a rather narrow range of the agarose concentrations, in nature it is likely that there are large gradients in the restriction of motility by the complexity of the matrix. If so, spreading through a semi-dense matrix using a combination of growth and occasional migrations, is a strategy, which ensures both fast invasion and stable occupation.

While the flagella mutant’s loss of satellites indicates the importance of flagella, more research is needed to explore how rare migratory events are enabled. For example, is it the inhomogeneity of gels that sometimes allows a cell to swim over long distances before being trapped? Or is it the cells that happen to express the relevant motility genes in high enough copy numbers to overcome the gel’s resistance? Revealing the origin of the stochasticity behind rare excursions, may highlight unknown mechanisms of robust invasion of bacteria in complex environments.

## Materials and methods

### Bacterial strains and culture media

The *Escherichia coli* strains used in this study are derivatives of the wild-type MG1655 (K-12 F-lambda- ilvG- rfb-50 rph-1) [62] and are all listed in Table 1 and detailed in ref. [63] and references therein. However, the non-flagellated Δ*fliC*, (Δ*fliC*::kan) mutant (NM109), was constructed specifically for this study through P1 transduction by moving Δ*fliC*::kan from JW1908-1 (Keio collection [64]) to MS613 followed by selection on kanamycin [65]. All strains were transformed with the plasmid pVS132 carrying an Isopropyl *β*-D-1-thiogalactopyranoside (IPTG) induced yellow fluorescent protein (YFP) and ampicillin resistance for selection [66] following a previously published protocol [67–69].

#### Culture media

Experiments were done using either of the two growth media: The rich Luria-Bertani (LB) composed of 1% tryptone, 0.5% NaCl and 0.5% yeast extract or M63 minimal media consisting of 20% 5 × M63 salt [70], 1 μg/ml B1, 2 × 10^−3^ M MgSO_4_, and supplemented with 20 μg/ml glucose (M63+glu). Both media were supplemented with 100 μg/ml ampicillin, unless otherwise stated.

### Growth rate measurements

*E. coli* strains were grown overnight at 37 °C while shaking in either LB or M63+glu medium, before diluting 1000× in fresh medium without antibiotics. Initial OD_600_ value (NanoPhotometer C40) was measured before incubation (37 °C) and repeated every hour.

### Swimming assay

Prior to experimentation, 96 mm dishes with 25 ml of either LB or M63+glu and various agarose concentrations: 0.2%, 0.3%, or 0.4% (w/v) were prepared and left to dry for 18 h on the bench. In parallel, strains were grown overnight while shaking (37 °C) in LB before inoculating 1 μl in the middle of each dish. After 24 h of incubation (37 °C), the range diameters were measured.

### 3D bacterial colonies

*E. coli* strains were grown overnight at 37 °C while shaking in LB medium (OD_600_ ≈ 4), before performing three consecutive thousand-fold dilutions until reaching a concentration of ≈10^3^ cells/ml. Then cells were embedded in a transparent semi-dense matrix using the following procedure: First, media with 0.2–0.6% (w/v) agarose (SERVA cat. no. 11404) was melted (in a microwave) and shaken rigorously to ensure homogeneity. To minimize evaporation (and heat-induced aging [71]), we opened new 25 ml bottles of media and agarose each time, and made sure to avoid boiling. Secondly, media with agarose was aliquoted into 1 ml tubes and placed in a block-heater at 55 °C. After approximately 20 min, when the mixture had reached 55 °C, it was supplemented with ampicillin (100 μg/ml) and 0.5 mM IPTG (for YFP induction). Then, 10 μl of the diluted overnight culture was added and the mixture was immediately—to prevent untimely gelification and heat shocks—poured into a petri dish (WillCo HBST-5040) with glass bottom. This results in a final concentration of ≈10 cells pr. well (i.e 10 cells/ml). After a few minutes the mixture had solidified and the well was incubated upside-down (37 °C) for 13 h (LB) or 15 h (M63 + glu) for all agarose concentrations: 0.2–0.6% (w/v). The different incubation times were chosen to balance different media-dependent growth rates (Supplementary Fig. S2). Using these small volumes, the matrix thickness was less than 400 μm, which ensures a minimum of oxygen depletion. To check this further, we compared the number of satellites pr. Colony with the position in the well (z-direction) and found no obvious correlation. However, the cost of this thin layer is a high risk of the colonies growing onto (and spreading fast over) the surface. Therefore, we discarded all wells, with colonies where this kind of growth had happened.

To parallel this assay, agarose was interchanged with 0.5% (w/v) agar (BD Difco Bacto Dehydrated Agar, Fischer Scientific) following the exact same procedure as described above. However, we found that the resulting agar gel is softer than the agarose gel, so, the density of agar 0.5% matches ~0.3% agarose (Supplementary Fig. S9).

### Imaging of 3D bacterial colonies

3D colonies in microwell dishes were imaged with a laser-scanning confocal microscope (LSCM) (Leica, SP5) and a 20× air immersion objective (Nplan,L20x,0.40corr ∞ ). YFP was excited by an argon laser with a wavelength of 514 nm and emission was collected around 550 ± 30 nm.

To estimate how the colony radius changes with time, colonies were imaged with an inverted Nikon Eclipse Ti fluorescent microscope (Nikon, Tokyo, Japan) using a 20x air immersion objective (Splan flour,L20x,0.45corr∞) paired with an Andor Neo camera (Andor, Belfast, UK). YFP was excited by a Hg lamp and the emission was collected at 535 ± 30 nm upon excitation at 500 ± 20 nm with a frame rate of one every 40 min.

The same approach was taken for the time-lapse movies of growing colonies (Supplementary Movie 1–5), however, the frame rate was one every 50 min. The reason we used wide-field fluorescence instead of LSCM for time-lapses was to reduce bleaching and photo-toxicity.

#### Morphology measurements

For snapshots of 3D colonies, we collected z-stacks that captured the entire colony with (*x*,*y*)-resolution of 1.52 μm/pixels and optimized z-resolution of 1.33 μm. The total imaging time of the colony was on the order of few minutes.

### Image processing

As the typical penetration depth of a LSCM is around 100 μm [72] and the dense colonies were of the order of 200 μm, the part of the colony furthest away in the scanning-laser direction (z-direction) suffers from strong distortions. Therefore, the following analysis was restricted to the half-colony closest to the LSCM objective by cropping the collected fluorescence z-stack using a custom-made Fiji [73] routine. 3D image segmentation was done with BiofilmQ [74] using the Otsu method for thresholding. Lastly, background noise was removed by eliminating outlier voxels of clusters smaller than 11 μm^3^. The following BiofilmQ parameters were exported for subsequent analysis: Convexity, number of satellites, volumes, distances between center-of-masses, and nearest-neighbour objects. The same BiofilmQ parameters were exported for the in silico colonies, the thresholding step was skipped for them (as they are binary per se).

### Modified Eden growth model

To reproduce the features of the obtained experimental results a modified Eden growth model was implemented. In a three-dimensional cubic lattice of linear system size *L*, each lattice site can be occupied by at most one bacterial cell. Each cell can grow at a rate *k* if there is at least one empty site among their six next nearest-neighbour sites. On top of this, if three or more of the next nearest sites are empty, then in addition to the growth, the cell can jump to another location. This is implemented as a jump to a new randomly chosen location that happens at a rate *k*_*s*_. This is implemented by counting the number of the cells that can only grow *N*_1_ and the number of cells that can both grow and swim *N*_2_ at each update and by applying a Gillespie algorithm. The initial condition is a single cell placed in the center of the system and then the following steps are iterated:

1. Find all the cells in the lattice and count the number of their empty nearest-neighbours. Among them, a cell that has less than four available neighbouring sites belongs to the population *N*_1_, otherwise, the cell belongs to the population *N*_2_.
2. Define an array containing all empty neighbouring sites, corresponding to the surface of the colony.
3. Compute the total event rate *T* = *N*_1_·*k* + *N*_2_·(*k*+*k*_*s*_), and determine the duration to the next event as *τ* = −ln(*r*)*/T*, where *r* is a random number from a uniform distribution (*r* = *U* ⊆ (0,1)). Proceed the time by *τ*.
4. Draw a random uniform number, *a*, from a uniform distribution between zero and one (a = *U* ⊆ (0,1)) and determine which event to happen by the following procedure:
  - If *a* > (*N*_1_ + *N*_2_)·*k/T* a growing event happens. Choose a random surface site and add a new cell to it.
  - Otherwise, a swimming event happens. Choose a random cell of the sub-population *N*_2_. Generate three random Gaussian distributed numbers of zero mean and standard deviation *σ*, to find the new position of the cell. If the new cell position is empty, update the cell position. If the new position is already occupied by another cell, draw a new position from the above-mentioned procedure until finding a new empty position.
5. Return to (1).

Pseudo-code is provided in the Supplementary text.

### Statistics

All mean values are given as (mean ± SD) unless stated otherwise and only when data are tested against the null hypothesis that it is normally distributed.

### Supplementary information

Supplementary movie 1

Supplementary movie 2

Supplementary movie 3

Supplementary movie 4

Supplementary movie 5

Supplementary figures

Supplementary code

## Data Availability

The code to generate the simulated data is available in Zenodo with 10.5281/zenodo.7414919.
