# Supplementary material for: Motility mediates satellite formation in confined biofilms
Source: ISME J. 2023 Aug 17;17(11):1819–27. doi: 10.1038/s41396-023-01494-x (PMC10579341; doi:10.1038/s41396-023-01494-x)
Supplement: Supplementary file 6 — Supplementary figures [file 41396_2023_1494_MOESM6_ESM.pdf]

Supplementary

Motility mediates satellite formation in confined biofilms

Mireia Cordero[1], Namiko Mitarai[1,*], and Liselotte Jauffred[1,*]

[1]The Niels Bohr Institute, University of Copenhagen, Blegdamsvej 17, DK-2100 Copenhagen O, Denmark
[*]Correspondence: mitarai@nbi.dk, jauffred@nbi.dk

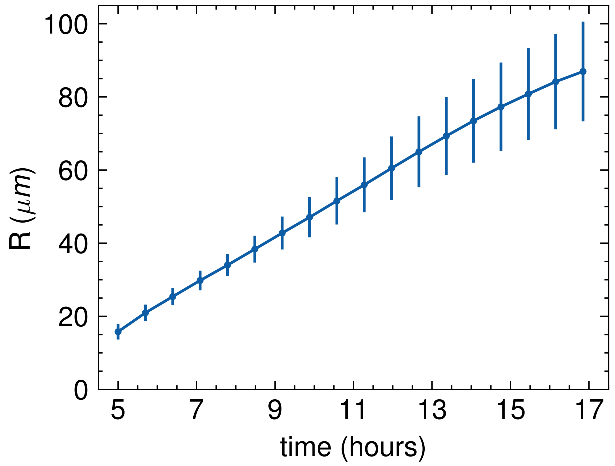

Figure S1: Colony growth dynamics for parental strain (wt). The colony radius (R) versus time in minimal medium (M63+glu) at intermediate agarose concentration (0.30%). The full line is the average development (N=8) and the error bars are ±SD.

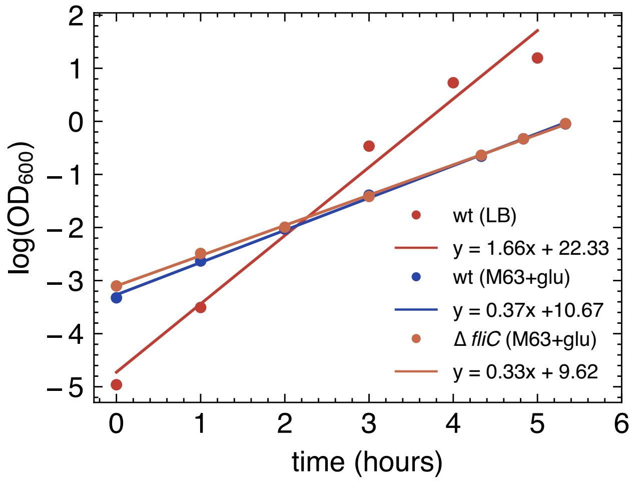

Figure S2: Growth curves for parental strain (wt) and flagella mutant (Δ*fliC*). Plot shows the experimental data (circles) and the exponential fit (full lines). The doubling time is found as the $ln(2)$ divided by the slope of the growth curve (full lines). The resulting doubling times are 27.8 min (wt, LB), 68.4 min (wt, M63+glu), and 72.8 min (Δ*fliC*, M63+glu).

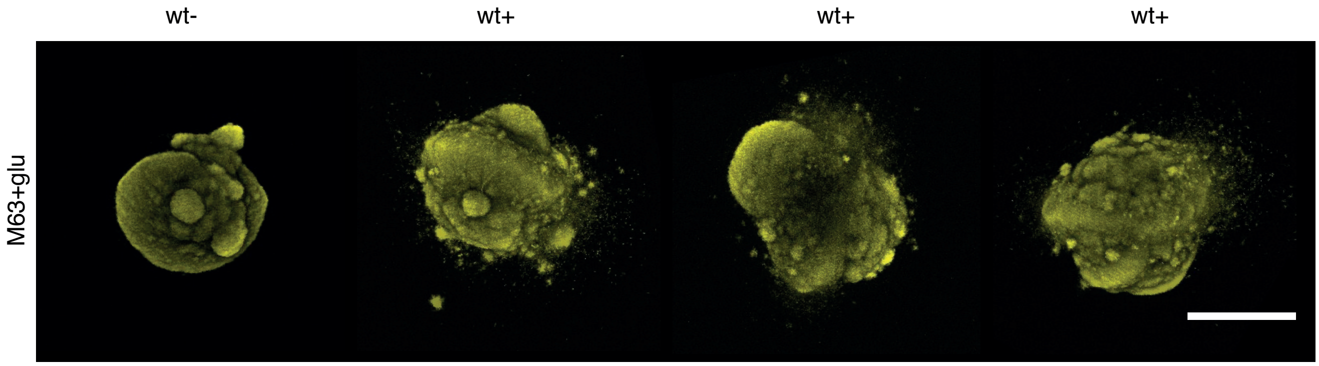

Figure S3: Satellite morphology in mature 3D colonies. Examples of pseudo-colored 3D colonies (maximum intensity projections) in 0.5% agar and minimal medium (M63+glu). These examples shows inhomogeneity of morphologies without satellites (wt−) or with satellites (wt+). The scale bar corresponds to 200 μm.

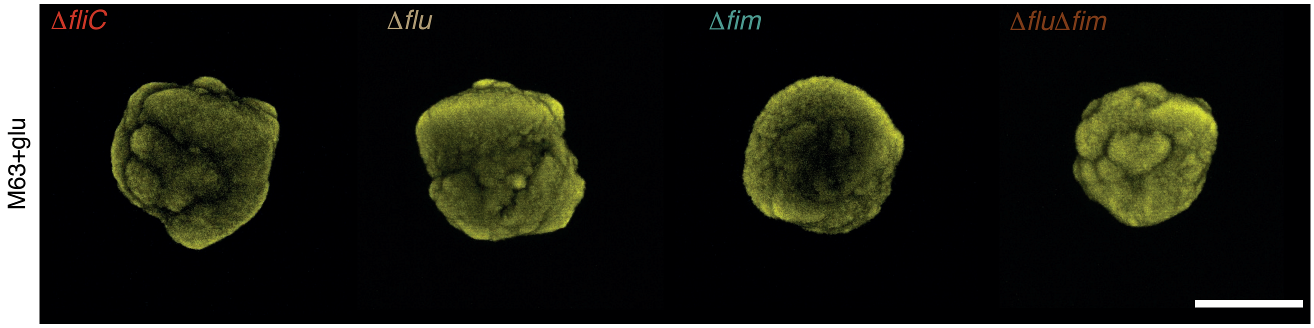

Figure S4: Morphology of mutant 3D colonies. Examples of pseudo-colored fluorescent colonies (maximum intensity projections) grown in M63+glu with 0.5% agar. The mutants lack either flagella (Δ*fliC*), antigen 43 (Δ*flu*), type I pili (Δ*fim*), and the double deletion (Δ*flu*Δ*fim*). Scale bar corresponds to 200 μm and color coding is the same as in figure 2A in the main article.

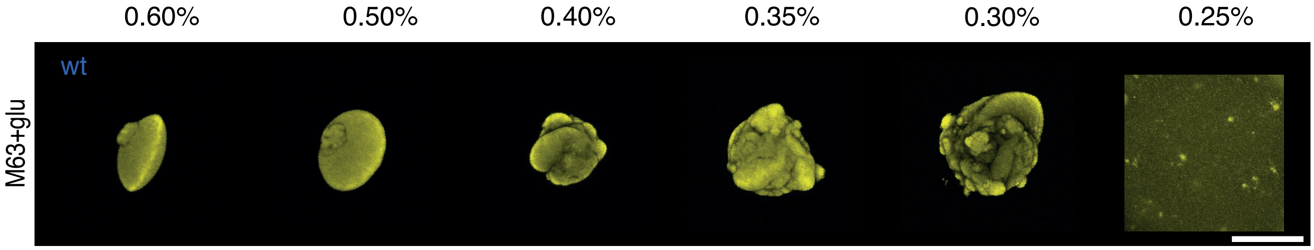

Figure S5: Effect of matrix elasticity on 3D biofilm morphology. Examples of pseudo-colored fluorescent wt biofilms (maximum intensity projections) grown in minimal medium (M63+glu) at various agarose concentrations. The scale bar corresponds to 200 μm. At low agarose concentration (0.25%) cells swims through the media and at high concentrations (> 0.50%) biofilms' have an oblate shape and a smooth surface.

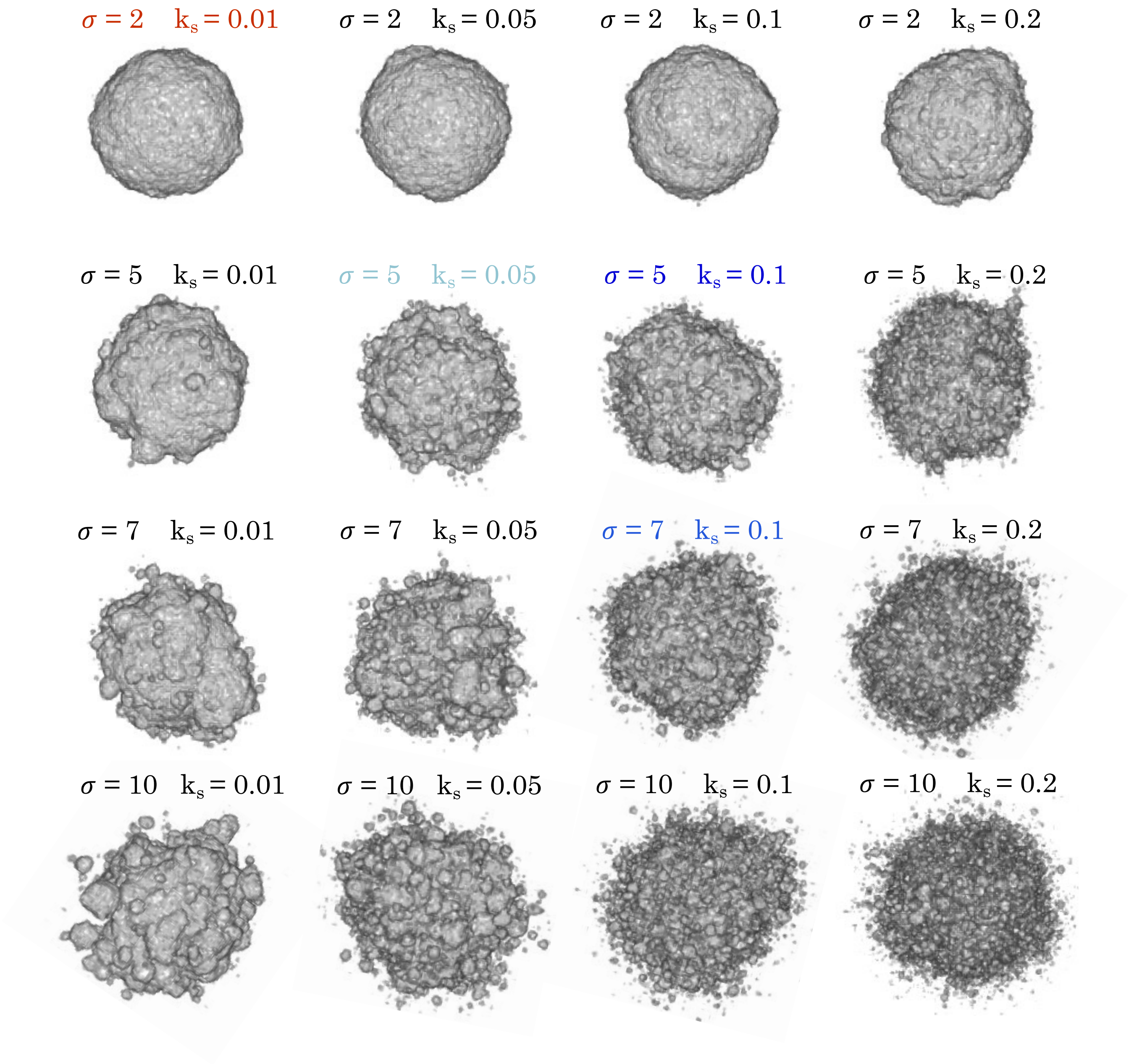

Figure S6: Effect of frequency, k_s, and distance, σ, of jumps in the simulations of 3D biofilms after 10^6 division/jump events. The color-coding is the same as in figure 4A in the main text.

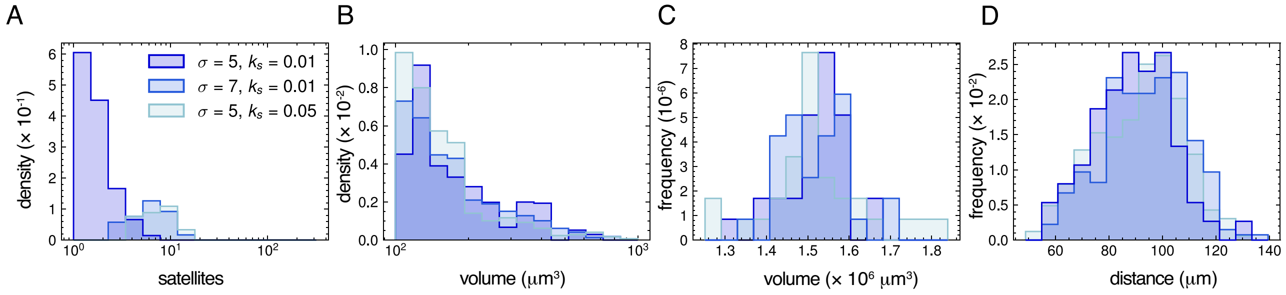

Figure S7: Satellite morphology in 3D biofilms. Simulated 3D biofilms (N=30) for 3 different sets of σ and k_s (N=30). Each lattice site corresponds to 1 μm and the color-coding is the same as in figure 4A in the main text. **B:** Distribution of of the number of satellites pr. biofilm (log-scale). **C:** Distributions of satellite volumes (log-scale) in either σ = 5 and k_s = 0.01 (N=61), σ= 7 and k$_s$ = 0.01 (N=223), and for σ= 5 and k$_s$ = 0.05 (N=271). **D:** Distribution of volumes of the main colonies. **E:** Distributions of distances from the center-of-mass of satellites to the center-of-mass of the main colony.

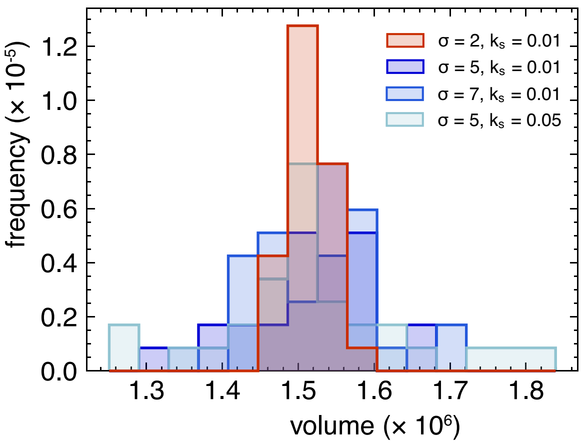

Figure S8: Total 3D biofilm volumes for simulated 3D colonies (N=30), where each lattice site corresponds to 1 μm. Color-coding is the same as in figure 4A in the main text.

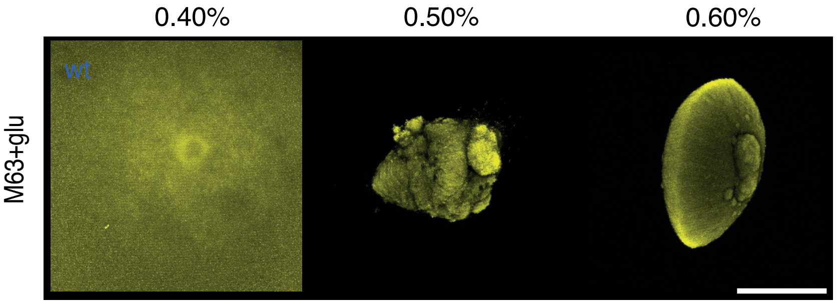

Figure S9: Effect of matrix elasticity on 3D biofilm morphology. Examples of pseudo-colored fluorescent wt biofilms (maximum intensity projections) grown in minimal medium (M63+glu) at various agar concentrations. The scale bar corresponds to 200 μm. At low agar concentration (0.40%) cells swims through the media and at high concentrations (> 0.60%) biofilms' have an oblate shape and a smooth surface.
