# Supplementary material for: Motility mediates satellite formation in confined biofilms
Source: ISME J. 2023 Aug 17;17(11):1819–27. doi: 10.1038/s41396-023-01494-x (PMC10579341; doi:10.1038/s41396-023-01494-x)
Supplement: Supplementary file 7 — Supplementary code [file 41396_2023_1494_MOESM7_ESM.pdf]

Computational Tools

Motility mediates satellite formation in confined biofilms

Mireia Cordero[1], Namiko Mitarai[1,*], and Liselotte Jauffred[1,*]

[1]The Niels Bohr Institute, University of Copenhagen, Blegdamsvej 17, DK-2100 Copenhagen O, Denmark
[*]Correspondence: mitarai@nbi.dk, jauffred@nbi.dk

Input: Lattice size L, growth rate k, swimming rate k_s, standard deviation std and an average μ=0 of a Gaussian distribution and the total number of events P
Result: Aggregate of cells in a 3D lattice

```
1  Generate a 3D lattice of dimension L with empty sites Lat(i, j, k) = 0 and occupied sites Lat(i, j, k) = 1;
2  Place a single cell in the center of the lattice

                         Lat (L/2, L/2, L/2) = 1

   for p ← 0 to P do
3      Define an empty colony surface, S;
4      Define an empty swimmers array, swim;
5      Set the number of each cell types, N1, N2 to zero;
6      foreach occupied site Lat(i, j, k) = 1 do
7          Find and count the number of empty near neighbours β

               Lat(i ± 1, j, k) = 0   Lat(i, j ± 1, k) = 0   Lat(i, j, k ± 1) = 0

           Add the available sites position to the surface of the colony S.
8          if β > 3 then
9              Add an element to N2 ;
10             Save the cell position in swim;
11         else if 0 < β ≤ 3 then
12             Add an element to N1 ;
13     end
14     Find the total event rate T = N1 · k + N2 · (k + k_s);
15     Determine which event happens at time t = −ln(r)/T, with r = U ⊆ (0, 1), by trowing a random number from an
         uniform distribution a = U ⊆ (0, 1) ;
16     if (N1 + N2) · k/T < a then
17         Chose a random surface site S(m, n, l) and update the colony by setting a cell in that position Lat(m, n, l) = 1;
18     else
19         Throw three Gaussian numbers to determine the final position of the cell: x_g = P(x)   y_g = P(y)   z_g = P(z);
20         Select an element of the swimmers array Lat(m′, n′, l′);
21         if Lat(m′ + x_g, n′ + y_g, l′ + z_g) = 0 then
22             Lat(m′, n′, l′) = 0;
23             Lat(m′ + x_g, n′ + y_g, l′ + z_g) = 1;
24         else
25             Go to 19;
26         end
27     end
28 end
```

Algorithm 1: 3D Eden Growth Model with swimmers
